# Editorial: Genotype-by-environment interaction in farm animals: from measuring to understanding

**DOI:** 10.3389/fgene.2023.1267334

**Published:** 2023-08-09

**Authors:** Eduard Murani, Hélène Gilbert, Wendy M. Rauw

**Affiliations:** ^1^ Institute of Genome Biology, Research Institute for Farm Animal Biology (FBN), Dummerstorf, Germany; ^2^ GenPhySE, INRAE, INP, Université de Toulouse, Castanet-Tolosan, France; ^3^ Departamento de Mejora Genética Animal, Instituto Nacional de Investigación y Tecnología Agraria y Alimentaria (INIA-CSIC), Madrid, Spain

**Keywords:** genotype-by-environment interactions, G×E, resilience, robustness, heat tolerance, CNV, pelvic prolapse, allele-specific expression (ASE)

There is increasing recognition of the need to study genotype by environment (G×E) interactions in farm animals. This interest is currently driven mainly by the desire to breed robust animals with high resilience and reduced environmental sensitivity (Rauw and Gomez-Raya, 2015), or by the search for genotypes adapted to specific environmental conditions, particularly heat (Passamonti et al., 2021). Knowledge of G×E not only provides the opportunity to find animals with superior ability to cope with environmental challenges, but also the prospect to better assess animal needs, which will benefit animal welfare and support precision livestock farming and medicine. With this Research Topic, we aimed to provide an overview of the current state-of-the-art of the genetic and genomic research covering all aspects, from the detection of G×E to the dissection of the underlying molecular mechanisms.

In the study of Bhatia et al. the authors performed a comprehensive investigation of the genetic basis of pelvic organ prolapse, a serious welfare and economic issue plaguing sow production. They found a moderate heritability of pelvic organ prolapse and six genomic regions, which together explain up to 9% of genetic variance of the trait in the studied population. Different incidence and heritability of pelvic organ prolapse reported in other cohorts provide a possible indication of G×E interaction influencing its manifestation, which warrants further investigation. As further discussed by the authors, more precise description of the prolapse condition is required for better understanding of the background of the trait.

It is not only important to thoroughly characterize the phenotypic trait, but also to accurately record and define the environmental conditions that will be investigated in terms of their interaction with the genotype. There is a steadily growing number of routinely measured parameters, e.g., from smart technologies, that can be harnessed for these purposes. Freitas et al. examined the best environmental descriptors based on seven climatic variables obtained from public weather station information as well as critical periods to evaluate heat tolerance and its genetic relationship to a range of reproduction, growth, and body composition traits in Large White pigs. They found evidence for G×E interactions for most of the studied performance traits. The authors further demonstrated that heat tolerance is heritable and genetically correlated with different performance traits, indicating that genetic improvement of heat tolerance in Large White pigs is feasible. Notwithstanding the observation that public weather station data show reasonable correlation with on-farm weather records, the authors recommend validation of their findings using in-barn recorded environmental data.

So far, most of the research dealing with G×E in livestock concentrated on detection and incorporation of G×E into breeding programs using quantitative genetic approaches (Tiezzi and Maltecca, 2022). Consequently, we have little knowledge on the molecular genetic background underlying G×E in livestock. Hu et al. investigated copy number variation (CNV) in three ecotypes of Tibetan sheep kept in three different environments differing primarily in altitude, feed availability and temperature. Because single nucleotide polymorphisms (SNPs) are ideal genetic markers for genome-wide association studies, other types of genetic variation are currently largely neglected. However, due to their higher functional potential and mechanisms linking their emergence with cellular responses to (challenging) environment, CNVs (Hull et al., 2017) and other types of genetic variations such as transposons (Pimpinelli and Piacentini, 2020) may play a major role in G×E interactions. Indeed, the CNVs identified by Hu et al. were enriched in genes related to relevant pathways including oxygen transport, and showed signs of selection in relevant candidates such as *RUNX1*. These results underscore the importance of considering different types of genetic variation in future G×E studies, as they are becoming increasingly accessible thanks to rapid advances in sequencing technologies and variant detection methods.

One of the major challenges of G×E studies is the need to measure phenotypes in large animal cohorts in different environments. This is not only laborious and costly, but is becoming increasingly problematic due to ethical concerns regarding animal welfare, particularly when considering stressful environmental conditions such as immune challenges. Murani and Hadlich explored G×E interactions in porcine immune cells by the analysis of allele specific expression depending on different *in vitro* stimuli (mimicking stress and infection) using mRNA-Seq data. This approach (termed condition-dependent allele specific expression, cd-ASE), originally developed in humans for high-throughput screening of G×E interactions (Moyerbrailean et al., 2016), combines power of the ASE analysis with the advantage of a well-defined environment *in vitro* cell models. Another benefit of the cd-ASE approach is that by detecting loci affected by environmentally sensitive regulatory variation it dissects the causal chain closer to its origin than genetic studies using organismal phenotypes. The authors found robust evidence of cd-ASE for several immune genes linked to animal health, demonstrating suitability of *in vitro* models combined with omics-based approaches as an alternative to quantitative genetic studies of G×E interactions on live animals.

We would like to thank all authors and reviewers for their valuable contribution to the Research Topic. The papers in the Research Topic provide a good cross-section through current research directions and challenges, and provide opportunities for future research. Current technological developments (e.g., in sensor technologies and omics techniques) and methodological advances (e.g., in big data analytics) will bring new possibilities for accurate and fine-grained characterization of the environment, phenotypic response and genome, and will provide a tremendous boost to research in this important area.
